# Integrating ethical and legal frameworks in multi-hazard disaster risk management

**DOI:** 10.12688/openreseurope.20093.2

**Published:** 2025-05-20

**Authors:** Zia Lennard

**Affiliations:** 1 R2M Solution SAS, Roquefort-les-Pins, France

**Keywords:** disaster risk management, decision support systems, multi-hazard, cascading impacts, legal frameworks, fundamental rights, ethical compliance, participatory action research

## Abstract

Contemporary disaster risk management (DRM) is increasingly challenged by the growing frequency and severity of natural hazards, exacerbated by climate change and complex societal interactions. Traditional approaches fall short in addressing interdependencies among multiple hazards and their cascading impacts. The European MEDiate project aims to develop an integrated Decision Support System (DSS) that effectively captures multi-hazard dynamics, emphasizing ethical compliance, fundamental human rights, and participatory engagement. This paper provides a comprehensive examination of the legal frameworks and fundamental rights considerations underpinning the development of the MEDiate DSS. Additionally, it explores diverse case studies across European regions to illustrate practical applications, current trends, ongoing challenges, and identifies opportunities for future research and innovation. Utilizing advanced methodologies, the DSS synthesizes real-time data with socio-economic indicators to deliver actionable insights for risk mitigation and adaptive response. Validated through extensive Participatory Action Research (PAR) across diverse European testbeds, MEDiate provides a flexible and resilient framework tailored to local and regional DRM contexts.

## 1. Introduction

Disaster Risk Management (DRM) faces growing challenges due to the increasing frequency, severity, and interconnectedness of hazards. Effective information exchange among stakeholders is crucial for robust disaster preparedness and response. Traditional risk assessment methodologies fail to account for these interdependencies, necessitating the development of an integrated framework capable of capturing and analyzing multiple hazards simultaneously. Decision support systems (DSS) for DRM are not one-size-fits-all; their frameworks and methodologies often differ by region, hazard type, and institutional context.

While this Open Letter primarily focuses on the rights enshrined in Articles 7 (respect for private and family life), 8 (protection of personal data), and 45 (freedom of movement) of the Charter of Fundamental Rights of the European Union, the broader integration of rights-based frameworks into Disaster Risk Reduction and Management (DRRM) must extend beyond this. Rights such as non-discrimination, the right to life, the highest attainable standard of physical and mental health, and the rights of persons with disabilities, children, and migrants are also central. Many of these are grounded in international human rights law and regional instruments like the European Social Charter. As MEDiate evolves, these rights will become increasingly important in shaping both design and operational decisions.

DSS vary widely depending on hazard type, regional needs, and institutional contexts. Understanding these variations can inform more effective system development. Geographic and institutional context also plays a role in how DSS are implemented, while systems like Global Disaster Alert and Coordination System (GDACS) provide a common operating picture for large, cross-border disasters, while regional systems focus on hazards with regional coherence and ensure interoperability among neighboring countries' data. National and local DSS are tailored to specific governance structures and needs, such as HAZUS in the United States for hazard scenarios, real-time public alerting and engineering controls in Japan, and community-centric DSS in Africa that incorporate local demographic data and participatory mapping of risks. Comparative evaluations suggest that no single framework is universally "best" – each has trade-offs. Multi-criteria DSS are praised for transparency and stakeholder inclusiveness, but they can be subjective and may not capture complex hazard interactions. Optimization-based DSS offer rigor and can handle complexity but may be computationally intensive and less interpretable to decision-makers without technical backgrounds. Simulation and scenario-based DSS provide rich insight into possible futures but require quality data and expert interpretation. The trend in many regions is toward hybrid systems that combine elements of these methodologies. Collaborative research and cross-border collaborations are helping propagate best practices, ensuring that advances in DSS methodology in one context can inform improvements elsewhere while respecting local needs and constraints.

As a member of the MEDiate project team responsible for ethical and legal oversight, this Open Letter reflects both internal insight and critical analysis of the system’s development. While this Open Letter primarily focuses on the rights enshrined in Articles 7, 8, and 45 of the Charter, the broader integration of rights-based frameworks into disaster risk reduction and management must extend beyond this—particularly toward non-discrimination, health, and the rights of persons with disabilities as recognized in international human rights law.

## 2. The MEDiate DSS for multi-hazard risk assessment and disaster response

The MEDiate European project focuses on multi-hazard risk assessment and disaster response, focusing on human rights, inclusive decision-making, and methodological rigor. The MEDiate DSS is a data-driven disaster risk management system that uses real-time data streams and demographic and infrastructural indicators to analyze interactions between hazards, vulnerabilities, and response mechanisms. The project uses empirical testing across multiple European testbeds to enhance local and regional disaster risk management in complex, resource-constrained environments. The DSS uses probabilistic modeling, network analysis, and exposure mapping to understand multi-hazard dynamics and incorporates adaptive learning algorithms for improved predictions. Validation is conducted through a Participatory Action Research methodology, including structured workshops with emergency responders, comparative analysis with existing DRM tools, and iterative testing across multiple testbeds.

The MEDiate DSS currently combines real-time and spatial data—such as hazard maps, transportation flows, and demographic indicators—to provide decision-makers with prioritized options. While certain analytic processes (e.g. hazard impact estimation or routing simulations) are automated, final decisions about actions like issuing evacuation alerts, mobilizing emergency services, or reallocating resources remain human-led. Future system iterations aim to support computer-assisted decision-making by clearly flagging options aligned with ethical priorities and legal standards, particularly where sensitive data is involved. Throughout, human oversight remains a core safeguard.

## 3. Frameworks for disaster risk information exchange

Global agreements like the WHO/PAHO on disaster coordination ^1^ and the Sendai Framework ^2^ emphasize data sharing and communication in DRM, encouraging early warning systems and knowledge-sharing. These frameworks do not impose legal obligations on member states but encourage integration into national policies. Section 3 focuses on legal DRM regimes, including international, European, and national.

### 3.1 European and cross-border information-sharing regimes

The EU Civil Protection Mechanism (UCPM) is a crucial mechanism in Europe for cooperative disaster response, enabling member states to exchange information on disaster risks and jointly prepare for emergencies. This framework facilitates rapid data sharing, requests for assistance, and resource pooling, facilitating mutual aid and creating legal clarity for cross-border data exchange in crises ^3^. The DRM Knowledge Centre (DRMKC) of the European Commission integrates scientific data and develops tools to translate complex risk information into usable knowledge for policymakers ^4^. International organizations like the United Nations Office for Disaster Risk Reduction (UNDRR) and the World Bank's Global Facility for Disaster Reduction and Recovery emphasize the need for robust, multi-dimensional strategies that integrate ethical principles, fundamental human rights, and inclusive governance.

Global databases like DesInventar ^5^ and platforms like UNDRR's Risk Information Exchange (RIX) ^6^ and the GDACS fill information and coordination gaps in the first hours after major disasters by aggregating alerts and impact estimates from around the world ^7^. These systems and tools set the backdrop for cross-border information exchange and highlight the importance of interoperability and open access, while showing how crossing organizational and national boundaries in information-sharing can save lives. European legal regimes also account for personal data protection even in the context of disaster response, with the EU General Data Protection Regulation (GDPR) establishing strict rules for processing personal data ^8^. Other EU laws indirectly support DRM information exchange, such as the INSPIRE Directive and the Floods Directive. Data protection standards involve specific measures such as encryption, anonymization, access controls, data minimization, and timely deletion of data to ensure privacy and security under GDPR.

### 3.2 National frameworks in MEDiate testbed countries

Section 3.2 describes how the MEDiate testbed countries operate within the European context but have their own national laws and regulations governing disaster risk information management. Data protection and privacy laws are common across all four countries, and authorities must adhere to GDPR principles for citizen data collection. However, differences in administrative structure and specific legislation can affect the modalities of information exchange. France's highly codified system (Civil Security Code and ORSEC plans) contrasts with the UK's multi-agency cooperation duties under the Civil Contingencies Act, yet both aim for a coordinated flow of information in crises. Norway and Iceland, being outside the EU but in the EEA, closely align with EU directives on data and civil protection; while also leveraging regional cooperation (e.g. the Nordic countries exchange crisis information through agreements and utilize EU’s Copernicus emergency satellite data). These national regimes create the legal environment for initial development of the MEDiate DSS, tailoring its data inputs/outputs and user access controls to each locale’s requirements.

***3.2.1 Norway.*** Norway's Personal Data Act, implementing GDPR, and sectoral laws for civil protection and emergency planning are crucial for ensuring data protection and collaboration. The country's Civil Protection Act (2010) mandates municipalities to conduct risk analyses and inform the public of hazards, establishing legal duties for information sharing among local authorities, the Norwegian Directorate for Civil Protection (DSB), and other agencies. The Norway testbed in Oslo focuses on multi-hazard scenarios like quick clay landslides, floods, and storms. Technical platforms like national map services and ArcGIS Online facilitate data exchange between municipal services, transportation authorities, and hazard mapping agencies. The legal framework allows agencies to share geospatial and sensor data under specific agreements, provided personal data is handled under the Personal Data Act. Norway's hazards include forest fires, quick clay landslides, floods, and storms. Agencies like the Norwegian Directorate for Civil Protection (DSB) and the Norwegian Water Resources and Energy Directorate (NVE) handle hazard mapping and emergency notifications. Platforms like AEP MAP-Service and AEP ArcGIS Online facilitate data sharing, while mobility and public transport data can also be factored into evacuation route planning. The Norwegian testbed, MEDiate, aims to enhance collaboration between municipalities, national agencies, and local communities by integrating hazard layers into a unified DSS interface. The system also incorporates fairness checks to avoid urban biases that might overlook remote or rural settlements.

***3.2.2 France.*** France's DRM governance is based on the Environmental Code and Civil Security Code, along with EU GDPR requirements. Local authorities, such as municipal governments in Nice and the Préfecture des Alpes-Maritimes, oversee emergency management. MEDiate's Disaster Risk Assessment System (DSS) aligns with existing frameworks like ORSEC, ensuring synergy with established early warning systems. France's extensive legal regime for disaster risk management and communication, exemplified in Nice, involves the Prefect and municipal authorities sharing responsibility for risk information dissemination. The French Civil Security Code regulates the assessment of major natural risks, such as floods, landslides, and wildfires. Nice's likely hazards include riverine and coastal flooding, landslides, extreme weather, heatwaves, forest fires, and earthquakes. Multiple information systems, such as Météo-France and networks like RAINPOL and PREDICT, provide real-time weather and flood forecasts. The challenge is integrating these flows, as all data sharing operates under French and EU law. MEDiate's DSS in Nice targets hazards such as flooding, landslides, extreme weather events, forest fires, and seismic activity. By interfacing with localized data and legal structures, MEDiate aims to unify hazard exposure modeling for floods, windstorms, and heatwaves under one multi-hazard system.

***3.2.3 UK.*** The Essex testbed in the UK faces a complex legal landscape involving data protection, emergency planning, water management, planning policy, and transparency. The UK's Civil Contingencies Act 2004 and the National Planning Policy Framework (NPPF 2021) are key legal instruments that require local authorities to share information and warn the public about potential risks. The integration of ethical considerations into evacuation planning—especially for persons in situations of vulnerability—is a significant legal and operational challenge. While the DSS can identify at-risk populations by combining datasets on age, disability, or geographic isolation, this is only the first step. The design of the DSS anticipates both computer-assisted and potentially fully automated decision pathways. Where personal data—especially special category data—is processed to recommend evacuation routes or prioritize assistance, the DSS includes safeguards such as access control, anonymization, and user accountability. However, automation must not obscure the ethical dimensions of displacement. For example, routing persons with disabilities to shelters without adequate facilities may breach their rights under the CRPD and Article 4 of the Charter (prohibition of inhuman or degrading treatment). As such, the MEDiate DSS is being developed with “human-in-the-loop” protocols to ensure oversight, traceability, and redress where needed. Ethical evacuation decisions must be informed by participatory input, uphold human dignity, and ensure minimum conditions in receiving facilities. This ethical architecture ensures that any DSS-supported limitations on freedom of movement are made only with human oversight, applying necessity and proportionality tests aligned with fundamental rights principles. The tension between freedom of movement (Article 45) and the duty to protect life is especially acute in this context. While the UN Guiding Principles on Internal Displacement allow for non-arbitrary evacuations in the face of imminent threats, recent work by Burson *et al.* (2018) highlights that state obligations must be carefully balanced with individual agency and procedural safeguards ^9^. The Essex testbed faces hazards such as pluvial flooding, fluvial flooding, coastal storm surges, droughts, and heatwaves. Sector-specific legislation, such as the Flood and Water Management Act 2010 and Flood Risk Regulations 2009, mandate local authorities to assess flood risks and publish hazard maps and risk management plans. The Land Drainage Act 1991 facilitates data exchange between councils and water authorities. The National Planning Policy Framework integrates climate resilience and disaster risk into spatial planning, requiring hazard information to flow into development control and public consultations. Freedom of Information laws allow citizens to request certain risk information. MEDiate's integrated hazard indicators and vulnerability analyses align with UK legislative environments, informing proactive DRM strategies. The testbed, centered on Essex County Council, deals with floods, heatwaves, and drought, requiring inter-regional cooperation. MEDiate's multi-hazard lens unifies the detection, prevention, and recovery phases of flood management, enabling rapid, ethically guided decisions on evacuations, resource allocations, and coordination across district and county lines.

***3.2.4 Iceland.*** Iceland's legal and institutional framework reflects its smaller population and centralized emergency management structure. The National Commissioner of the Icelandic Police leads disaster risk management, while the Icelandic Meteorological Office (IMO) provides hazard monitoring and assessment. The municipality of Múlaþing is legally responsible for communicating hazard information to its inhabitants and relaying warnings from national authorities when necessary. Iceland has implemented GDPR through a national Personal Data Act, ensuring that any handling of personal data for disaster management is done with privacy protection. The typical hazards in the Múlaþing area, such as mudflows, avalanches, storms, floods, and tsunamis, often require rapid dissemination of warnings. The legal framework supports this by giving clear authority to the police/Civil Protection Department to issue emergency messages while requiring coordination with municipal officials and technical experts like IMO. The MEDiate DSS in Iceland can leverage this clarity by feeding real-time monitoring data from the IMO and using it by local police and municipal leaders to decide when and how to alert communities. MEDiate effectively adapts to Icelandic regulations and local contexts, addressing specific physical and socio-economic vulnerabilities.

### 3.3 Technical interoperability and legal compliance

Interoperable technologies can enhance information sharing and support legal compliance in DRM. This involves establishing common data formats, APIs, and secure communication channels for quick data exchange during emergencies. Tools like GIS, remote sensing, and crowd-sourcing platforms are essential for DRM. The European Commission advocates for smart technology for better governance and real-time partnerships. The MEDiate testbed uses technical systems to fulfill legal mandates, such as aggregating data from various sources to provide a common operational picture. This automates data integration, ensuring no time is lost in manually exchanging emails or reports. Technical design can also embed legal safeguards, such as access controls and user permission levels, audit logs, encryption, and cybersecurity measures. Technology also improves public-facing communication, which has legal and safety implications. Modern DSS tools can automatically generate public alert messages when certain risk thresholds are crossed, helping authorities meet their communication duties. Technical interoperability between DSS and public alerting tools directly influences citizens' safety by ensuring coherent messaging and avoiding confusion. Investing in these technical linkages is an investment in both legal compliance and public trust, as citizens are more likely to heed warnings when messages are consistent and clear from an authoritative system, improving overall emergency outcomes.

### 3.4 Impacts on safety, planning, and policy

The legal frameworks governing DRM information exchange can significantly impact citizens' sense of safety and the effectiveness of local governance. When stakeholders share information fluidly and lawfully, it creates a more transparent and responsive disaster risk governance. This is expected to enhance public confidence in disaster risk governance. For example, in Iceland's small communities, knowing that the municipality and national agencies have a direct line of communication about avalanche or landslide risks – potentially via a shared DSS – can reassure residents that they will be alerted in time and that no warning signs will be overlooked.

Enhanced information exchange also feeds into better local planning and services. With access to richer risk data, local governments can incorporate multi-hazard insights into urban planning, infrastructure development, and emergency planning. The MEDiate project explicitly aims to support “disaster preparedness and long-term planning in areas where access to services, funding, and information is limited”. From a hazard communication standpoint, the combination of clear legal mandates and a DSS enables more effective public outreach and education, which further improves community safety. In several countries, there is movement towards recognizing the right of the public to be informed of risks. MEDiate’s testbeds can leverage the DSS to create more engaging communication, such as producing easy-to-understand risk maps or simulation results that can be shared at town hall meetings or on city websites.

At the local level, policies might be adapted to institutionalize the gains from MEDiate. A city government might update its emergency response plan to formally include the use of the DSS for information management, effectively making the tool and its processes part of the standard operating procedure. Regionally or nationally, the success of a multi-hazard DSS in these testbeds could encourage wider adoption, leading to national policy support (funding, training, standards) for such systems. Lessons learned from projects like MEDiate could shape future EU civil protection guidelines or even legislation on disaster-resilient digital infrastructures. In summary, the legal frameworks governing DRM information exchange are both an enabler and a work-in-progress; projects like MEDiate test their robustness and can catalyze improvements that ensure laws keep pace with technological advancement and evolving societal expectations for safety.

## 4. Fundamental rights and implementing the MEDiate Decision Support System

Section 4 examines fundamental rights in the context of implementing the MEDiate DSS following the project lifecycle. Ethical oversight includes mechanisms to ensure transparency, accountability, and fairness in how data is used and how DSS-driven decisions affect communities, ensuring no discriminatory impacts occur. Exemplified by Sendai, international DRM frameworks are increasingly incorporating human rights principles to emphasize the importance of protecting people and their assets while promoting human rights, including the right to development. It emphasizes empowerment and inclusive participation in DRM, particularly for those disproportionately affected by disasters. UNDRR has reinforced these principles, urging a human rights-based approach to DRM in line with international human rights law.

The World Bank and affiliated global initiatives have also adopted inclusive DRM policies that reflect human rights values. The World Bank's Environmental and Social Framework (2018) incorporates principles of transparency, accountability, participation, and non-discrimination in all projects, guiding its approach to avoiding harm and ensuring fairness in disaster-related investments. By addressing the needs of populations in situations of vulnerability and involving them in planning, these international frameworks and policies uphold human rights principles of equality and participation in DRM initiatives. Such global DRM frameworks (Sendai, UNDRR guidelines, World Bank policies) increasingly converge on a people-centered, rights-based paradigm, recognizing that resilience is fundamentally linked to human dignity and social equity. Promoting opportunities, abilities, and dignity for marginalized groups is crucial in DRM policies and programs. Addressing the needs of all population groups is essential for reducing disaster risks. Failure to address vulnerability can result in significant social and economic costs, as seen in the 2015 Nepal earthquake where 22% of women became economically inactive ^10, 11^.

Any DSS used for DRM must operate in compliance with fundamental rights, particularly when it processes personal data or influences actions that affect individuals’ liberties. The EU Charter of Fundamental Rights guarantees the rights to privacy (Article 7), protection of personal data (Article 8), and free movement (Article 45) among others ^12^. In the context of the MEDiate DSS – a tool that aggregates risk information and may guide decisions like evacuations or resource deployment – these rights provide a lens to evaluate its design and use. The following sections examine how the MEDiate DSS aligns with privacy and data protection norms (especially GDPR), what technical and organizational safeguards ensure individuals’ rights and freedoms are respected, and how the system might impact or uphold the free movement of persons in disaster scenarios. Within the MEDiate DSS, these principles operationally translate into role-based user permissions, algorithm transparency checks, and structured ethical reviews during decision-making processes.

### 4.1 Data privacy and personal data protection

Data privacy is crucial in disaster management and crisis operations, and systems must comply with regulations like the EU's GDPR when handling personal or community data. Balancing public safety with individual rights was challenging during the COVID-19 pandemic, as sharing detailed personal data could save lives but expose sensitive information. To achieve this, a DSS for DRM must incorporate strict data protection measures, robust governance policies, and ethical data practices. Transparency about data collection and usage, as well as accountability, are essential to ensure privacy is not eroded by crisis management needs. Adhering to frameworks like GDPR will not only meet legal requirements but also build public trust in DSS recommendations.

The MEDiate DSS, which handles multi-source data, must adhere to the GDPR and corresponding national data protection laws in each testbed country. GDPR is not just a bureaucratic hurdle; it embodies a fundamental right recognized in EU law – that individuals have control over their personal information and assurance that it will be used lawfully and securely. Any personal data processed by the DSS must have a valid legal basis (such as consent, vital interest of the data subject, or public interest in disaster management) and be limited to what is necessary for the specified purpose (data minimization). Sensitive personal data should be avoided unless critical and even then, handled with higher safeguards.

Mediate's hazard modeling and vulnerability assessments can largely run on aggregated or anonymized data, such as population counts, building values, or indices of social vulnerability. Implementing privacy by design involves features like role-based access, masking or aggregating data in public-facing visualizations, and automatic deletion of personal data after it is no longer needed for the disaster event or analysis. All four testbed countries have institutions and legal instruments to enforce these privacy standards. For MEDiate DSS implementation, an internal data management plan will likely define who is the data controller (probably the authority operating the DSS) and who are data processors (perhaps project partners managing the software or cloud). Data processing agreements would need to be in place among the project consortium to cover any exchange of personal data. Transparency is still key for trust, as the public should know that any personal information gathered for DRM is handled lawfully and not retained indefinitely or misused.

### 4.2 Security, resilience and free movement

The security of the Disaster Risk Management System (DSS) and its data is crucial for safeguarding citizens' rights and freedoms. Unreliable or insecure systems can lead to false alarms, endangering lives, and undermining the right to security. The EU's Network and Information Systems (NIS) Directive emphasizes that disruptions in network/information systems can have societal effects and even impede the movement of people across borders. In a disaster context, an outage of a DRM DSS in one country might affect neighboring countries, leaving others blind to a creeping threat. Therefore, ensuring high availability, integrity, and cybersecurity of the MEDiate DSS is not only a technical requirement but a fundamental part of safeguarding the community's rights.

The NIS Directive and its successor (NIS2) encourage Member States to classify such systems and apply rigorous security measures. Even if the MEDiate DSS is not formally an "essential service" yet, its functions are part of critical infrastructure. Following NIS guidance, such as risk assessment, incident reporting, and cooperation with national Computer Security Incident Response Teams (CSIRTs), would be prudent. This includes strong encryption of data in transit and at rest, authentication protocols for users, firewalls and intrusion detection on the system's servers, and failover plans. Organizationally, regular security audits and drills can ensure the system's resilience.

A robust and secure DSS indirectly supports the free movement of persons, another fundamental value in the EU. A well-informed response, aided by a DSS, can minimize unnecessary travel restrictions and ensure that any limitations on movement are proportionate and brief. By including mobility and transport data, the DSS can help target warnings to travelers, which is important for free movement and tourism. From a rights perspective, any measure that the DSS recommends which could limit movement should be based on solid data and risk analysis to ensure it's truly necessary. The MEDiate DSS, by providing evidence-based risk assessments, can inform such decisions, balancing individual freedoms with collective safety.

### 4.3 Safeguards and ethical oversight

Implementing a Disaster Risk Management System (DSS) in line with fundamental rights involves not only software but also organizational safeguards and ethical oversight. EU-funded projects like MEDiate are required to address ethics and data protection from the start, including dedicated work on legal/ethical compliance, appointing an ethics advisor or board, and delivering on requirements such as an Ethics Deliverable or Data Management Plan. In MEDiate, these instruments ensure that data subjects' rights and freedoms are central considerations in system design.

Stakeholder engagement and inclusive governance are crucial ethical requirements for DRM tools. Engaging diverse stakeholders in planning ensures disaster policies reflect collective values and avoid discrimination. Participatory mechanisms, such as consulting local officials and NGOs, uphold procedural fairness and yield more culturally appropriate outcomes ^13^. MEDiate's participatory co-design approach helps mitigate this risk by incorporating diverse stakeholder input, while ethical oversight guards against any infringement of the principle of equal dignity.

On the data side, organizational measures such as training staff in data protection, having a clear process for individuals to query or correct their data, and appointing a Data Protection Officer for the authority using the DSS are important. Each testbed authority should integrate the DSS into their existing GDPR compliance structure, updating their public privacy notices to mention the DSS and its purposes.

Consent and community engagement are another safeguard area. If the DSS includes optional public-facing components, obtaining informed consent and giving users control is key. The Sendai Framework emphasizes people-centered approaches, empowering citizens with information while respecting their privacy choices. As the MEDiate DSS is designed to work in multiple countries, interoperability of legal norms is essential. International guidelines for disaster data sharing that include harmonized privacy and ethics rules help facilitate the MEDiate consortium's work and ensure that fundamental rights travel with the data.

### 4.4 Promoting trust and future policy directions

The MEDiate DSS, a disaster risk management system, is based on the fundamental rights principle of trust. Citizens need to trust that technologies used to protect them will respect their rights, which can lead to increased cooperation with authorities during emergencies. Trust in the system can be built through public communication about its capabilities and its responsibility in handling personal data. Local authorities can inform residents about the system's capabilities and clarify that it does not surveil or collect personal details. This can increase the community's sense of safety and reduce anxiety.

MEDiate's approach of democratizing access to scenario-based tools and bridging knowledge gaps is aimed at empowering communities, which is linked to fundamental rights such as the right to information and participation in societal efforts to reduce risk. By involving community representatives in scenario planning, the project acknowledges citizens as stakeholders and enhances legitimacy and compliance with disaster measures.

The successful integration of fundamental rights into a DRM DSS can influence broader legislative or policy developments. One potential evolution is the formal recognition of "data for disaster management" as a category warranting special attention. Policymakers might consider creating explicit legal provisions that clarify how personal data can be used in disasters, streamlining GDPR's existing clauses into a more straightforward emergency data-sharing protocol. Standardizing ethical requirements for AI or decision-support in the public sector is another area that could benefit from MEDiate's work.

From a free movement standpoint, more formalized cross-border cooperation agreements may be seen, allowing the flow of not just data but also people during emergencies. The EU Civil Protection Mechanism ^14^ already facilitates this, but fundamental rights considerations like handling personal data of foreign citizens or respecting the rights of evacuees crossing into another country might drive further refinement. A future EU guideline could detail how to manage transboundary evacuation operations, ensuring individuals maintain their rights while moving for safety.

## 5. Further case studies, challenges, and emerging trends

Real-world cases since 2019 demonstrate how DRM frameworks can be implemented in ways that uphold human rights and social equity. Section 5.1 presents a few illustrative examples spanning hazard types, climate-related disasters, pandemics, and humanitarian crises. The case studies were selected to showcase practical implementations of rights-based DRM approaches. Meanwhile, Section 5.2 touches on key challenges of implementing DSS for DRM, and Section 5.3 describes how DRM is rapidly evolving with new technologies and methodologies that aim to enhance decision support while promoting inclusivity and transparency.

### 5.1 Case studies of rights-based DRM implementation (2019–Present)

Academic literature categorizes rights-based DRM mplementation approaches by the decision methodologies they employ. Frameworks range from multi-criteria decision-making methods to mathematical optimization models and hybrid techniques. Some systems prioritize solutions by scoring alternatives against weighted criteria, such as the Analytic Hierarchy Process (AHP) or the Best-Worst Method, which has been applied in regions where stakeholder preferences and local knowledge must be factored explicitly into the prioritization of communities for evacuation or resource distribution.

Hazard contexts inspire different DSS methodologies. For instance, the European Flood Awareness System (EFAS) emphasizes continent-wide early warning and scenario modeling systems ^15^. EFAS is relevant because it demonstrates effective cross-border collaboration essential for MEDiate’s transboundary data-sharing needs. In drought and food security management, systems like the Famine Early Warning Systems Network (FEWS NET) have been established to aggregate climate data, crop forecasts, and socio-economic indicators into decision support for aid agencies. FEWS NET's integration of climate forecasts and socio-economic indicators offers insights for MEDiate’s adaptive learning algorithms.

DSS for rights-based DRM help to process data and present actionable insights, reducing the time needed for critical decisions on resource deployment. Case studies like the 2017 Oroville Dam crisis in California and India's post-disaster DSS for resource allocation have validated these benefits. Multi-hazard platforms like the Global Disaster Information System (GDACS) have proven instrumental in major crises, providing rapid impact estimates, early warnings, anticipatory action, and streamlined international coordination.

The COVID-19 pandemic highlighted the need to incorporate human rights into disaster management, with international organizations advising against betraying rights through public health measures. Inclusive governance was effective in fighting COVID-19, ensuring evidence-based policies ^16^. The Building Resilience through Inclusive and Climate-Adaptive DRR (BRDR) program in the Asia-Pacific prioritizes gender equality and rights-based approaches in disaster risk reduction. Tools like the Framework for Integrating Rights and Equality (FIRE), developed under the Building Resilience through Inclusive and Climate-Adaptative Disaster Risk Reduction (BRDR) programme, guide risk assessments and land-use planning by considering the needs of women, persons with disabilities, and other at-risk groups. FIRE comprises six dimensions: fundamental rights and equality, non-discrimination, participation and access to information, governance systems and structures, social norms and context, and agency and empowerment. These were synthesized from international law and operational guidance such as the Sphere Handbook17 and IASC Guidelines18. Applying FIRE in MEDiate would help align DSS design with international best practices for equitable disaster response ( https://rwi.lu.se/fire) ^19^. Communities are engaged in identifying hazards and shaping emergency preparedness plans, ensuring their voices guide the process.

The Cyclone Idai recovery in Mozambique (2019–2024) focused on human rights and inclusion, particularly for displaced and persons with disabilities. A consortium including the Mozambican government, the World Bank, and NGOs like Humanity & Inclusion (HI) implemented an inclusive rebuild program in hard-hit communities, constructing new housing for survivors with a focus on accessibility and dignity. HI teams conducted accessibility audits of infrastructure and provided input to adapt schools, clinics, and homes to meet universal design standards. The project also trained 200+ community members and humanitarian staff on inclusive practices, reinforcing the rights to adequate housing and equality for survivors in situations of vulnerability ^20^.

These case studies demonstrate that applying human rights principles in disaster contexts is feasible and beneficial. Approaches that uphold dignity, non-discrimination, and participation through inclusive planning, targeted protections for people in situations of vulnerability, and community engagement have resulted in more just and effective outcomes.

### 5.2 Key challenges in DSS integration and implementation

Despite significant advancements, several challenges persist in the development and implementation of DSS for multi-hazard DRM. One key issue is data interoperability, where disparate datasets from different agencies and sources often lack standardization. Ensuring seamless integration of diverse data streams remains an ongoing research priority. Another limitation is the computational cost of high-resolution simulations, which can limit real-time applicability in resource-constrained settings. DSS frameworks need to evolve to incorporate climate adaptation strategies dynamically, given the increasing frequency and intensity of climate-induced disasters. Future research must explore the use of hybrid AI models that combine deep learning with expert-driven rule-based systems to enhance predictive reliability and decision support.

Data integration and interoperability issues are among the most cited barriers in implementing DSS for multi-hazard management. A multi-hazard DSS must pull together information from numerous sources, such as meteorological data, seismic sensors, social datasets, and infrastructure networks, which often exist in incompatible formats and siloed systems. Studies highlight that many failures in disaster coordination stem from different agencies and systems being unable to seamlessly share data and models. Without interoperability, crucial information can fall through the cracks, undermining the very purpose of a DSS. Data quality and reliability present concerns, as incoming streams may be incomplete, outdated, or incorrect, especially in chaotic disaster conditions. DSS developers must grapple with uncertainties and ensure the system can function with imperfect data. There is also the risk of information overload: a comprehensive DSS might flood decision-makers with more data and model outputs than they can interpret under time pressure. Machine learning can filter out "unrelated data" and flag the truly relevant signals, mitigating cognitive overload during crises ^21^.

Another set of challenges lies in the ethical and social domain. As AI-driven and data-intensive DSS become commonplace, they raise questions about bias, transparency, and accountability. Algorithms trained on historical data may inadvertently perpetuate biases, such as underestimating risks to under-served communities if those areas were historically under-reported. The often "black box" nature of advanced AI models can make it unclear how a DSS arrived at a given suggestion, creating a transparency problem ^22^.

Practical implementation barriers can impede DSS deployment even when technical issues are solved. Resource constraints are a common hurdle, as developing and maintaining a sophisticated DSS requires funding, skilled personnel, and infrastructure. Interdisciplinary and inter-agency coordination is another practical challenge, as a multi-hazard DSS often requires collaboration between meteorologists, engineers, planners, first responders, government officials, and communities. Developers must address human-factor issues like interface design, training programs, and change management to make DSS work in practice.

### 5.3 Emerging trends and innovations in DRM Decision Support Systems

Technological innovations, such as artificial intelligence (AI) and blockchain, have significantly accelerated the capabilities of DSS for DRM, making them indispensable for real-time data processing, risk modeling, and scenario-based decision-making. However, the efficacy of these tools depends on their ethical and legal foundations, such as data privacy, algorithmic fairness, and stakeholder inclusion. An "ethics-by-design" approach to DRM involves embedding ethical considerations into the system development lifecycle, incorporating safeguards, and addressing legal requirements like GDPR. This approach maintains public trust and ensures technology-driven disaster management does not compromise human rights.

AI is being harnessed to improve risk assessment and early warning, with AI-assisted emergency response plans being developed by UN-led initiatives. AI is being used to quickly analyze multi-hazard data while maintaining transparency and bias mitigation. Pilot applications include predictive analytics for flood forecasting, computer vision for damage assessment via satellite imagery, and optimization algorithms for relief logistics. These tools can greatly improve the speed and accuracy of decisions, but human oversight and fairness criteria must guide their deployment ^23^.

Blockchain technology is being used to enhance trust and data integrity in DRM. It provides a decentralized, tamper-proof ledger for critical data like resource inventories, aid distribution, and identity records. Studies during the COVID-19 era suggest that blockchain can build trust, transparency, and accountability in complex disaster operations. Projects have used blockchain to track aid funds and maintain secure medical data exchanges during disasters ^24, 25^.

Geospatial technology and open data are enabling more inclusive, bottom-up risk mapping through tools like OpenStreetMap, mobile data collection apps, and participatory GIS platforms. These tools allow local communities to contribute directly to mapping their environment and hazards, improving data availability for early warning systems and capturing community knowledge in risk models. This approach fosters inclusivity, transparency, and public engagement, leading to more effective local risk reduction measures.

Comprehensive DRM platforms that integrate multiple hazard models, big data analytics, and user-friendly interfaces for decision-makers are emerging trends that promote transparency and inclusive governance. As these innovations mature, they are likely to become integral to how we approach multi-hazard risk management in an inclusive manner.

## 6. Recommendations and future directions

To truly be people-centered, MEDiate’s DSS should facilitate meaningful stakeholder engagement not only in its development but also in its deployment. Building on the PAR approach already used in testbeds, MEDiate can set up formal mechanisms for participatory governance. One approach is to establish local stakeholder committees in each pilot region (including community representatives, NGOs, and civic leaders) who regularly interact with the DSS team. These committees could co-design scenario assumptions, validate data inputs, and discuss the DSS’s recommendations, ensuring local knowledge and values are incorporated. Establishing regular participatory workshops for scenario validation involving local stakeholders and emergency managers and organizing mandatory comprehensive training in GDPR compliance, data protection practices, and ethical principles for all DSS users and administrators would be beneficial for technology adoption. As noted in disaster ethics research, involving community stakeholders leads to more fair, culturally appropriate decisions.

Future research extending beyond the scope of MEDiate should focus on implementing standardized data-sharing protocols and interoperability guidelines across DRM platforms. Additionally, ethical considerations, such as bias in predictive models and privacy concerns in data collection, require further investigation. Research could be focused on AI-driven DSS to improve computational efficiency, enhance real-time data integration, and increase explainability of decision-making algorithms.

The integration of blockchain technology for secure, decentralized data sharing is another promising avenue that could address trust and data integrity challenges possibly leading to tamper-proof data exchanges and enhancing trust in DRM practices. AI for risk assessment tools may be useful but can inadvertently reproduce societal biases in training data, leading to unfair outcomes. This raises equity issues in disaster response. To address this, ethical DRM DSS require bias mitigation measures, such as auditing algorithms for biased patterns, using diverse datasets, and applying fairness metrics. Ongoing monitoring and transparent validation are recommended to detect and correct any unfair impacts. This might involve algorithmic impact assessments or incorporating ethical AI frameworks that uphold principles of justice and respect for human rights. Ensuring explainability of AI decisions is crucial for stakeholder understanding and challenge ^26^. Moreover, explainable AI (xAI) technology can generate synthetic data to enhance predictive models, aiding in disaster risk identification. However, ethical concerns arise regarding the accuracy of synthetic data in models and concerns about data privacy and security. The use of synthetic data in predictive models may not accurately reflect real-world conditions, and its application in disaster response raises questions about data security ^27^. Ensuring long-term ethical adherence and privacy protection could be accomplished with data anonymization techniques including aggregation and pseudonymization of sensitive information and algorithm transparency checks through regular fairness audits, impact assessments, and xAI integration. Organizational mechanisms, such as appointing dedicated Data Protection Officers (DPOs) and Ethics Advisory Boards, overseeing compliance and ethical practices should be imperative for widespread MEDiate DSS adoption.

Looking ahead, the integration of broader fundamental rights—particularly social and economic rights—will be a focus in refining the DSS. This includes embedding frameworks like FIRE to assess both technical outputs and their ethical implications. MEDiate will also explore participatory protocols to ensure that automated recommendations do not replace community input, especially in decisions affecting relocation, access to services, and prioritization of assistance. Ensuring transparency in decision logic and human accountability remains a guiding principle.

## 7. Conclusion

Sustaining and enhancing MEDiate’s DSS is critical for long-term disaster resilience and sustainability, as it ensures the preservation of ethical and human rights principles, prevents discontinuity of research projects, and fosters institutional memory and capacity building among local authorities, civil protection agencies, and community stakeholders. This fosters fluency in MEDiate's modeling techniques, ethical constraints, and scenario-planning capabilities, which can then be transferred to other regions or institutions. The MEDiate DSS is also versatile multi-hazard preparedness, as it adapts to new threats through software updates, new data streams, and stakeholder feedback. This flexibility positions MEDiate as a living system, constantly updated to address emergent risks like pandemics, wildfires, or extreme weather events.

## Disclaimer

The views expressed in this article are those of the author. Publication in Open Research Europe does not imply endorsement by the European Commission.

## Ethics and consent

Ethical approval and consent were not required.

## Data Availability

No data are associated with this article.
