# Supplementary material for: Integrating ethical and legal frameworks in multi-hazard disaster risk management
Source: Open Res Eur. 2025 May 20;5:97. Originally published 2025 Apr 4. [Version 2] doi: 10.12688/openreseurope.20093.2 (PMC12171713; doi:10.12688/openreseurope.20093.2)
Supplement: Peer review history

# Reviewer report: Reviewer response for version 2

**DOI:** 10.21956/openreseurope.22091.r54417

**Published:** 2025-06-16

**Authors:** Matthew Scott

**Affiliations:** 1 Faculty of Law, Lund University, Lund, Skåne County, Sweden; 2 The Raoul Wallenberg Institute of Human Rights and Humanitarian Law, Lund, Sweden

Happy to see proposed improvement integrated.  The manuscript is okay to be accepted.

Where applicable, are recommendations and next steps explained clearly for others to follow? (Please consider whether others in the research community would be able to implement guidelines or recommendations and/or constructively engage in the debate)

Yes

Does the article adequately reference differing views and opinions?

Yes

Are all factual statements correct, and are statements and arguments made adequately supported by citations?

No

Is the rationale for the Open Letter provided in sufficient detail? (Please consider whether existing challenges in the field are outlined clearly and whether the purpose of the letter is explained)

Yes

Is the Open Letter written in accessible language? (Please consider whether all subject-specific terms, concepts and abbreviations are explained)

Yes

Reviewer Expertise:

International human rights law; human rights-based approaches to climate change adaptation and disaster risk reduction and management; all forms of environmental human mobility, including evacuation, planned relocation, displacement, forced evictions, resettlement, etc.

I confirm that I have read this submission and believe that I have an appropriate level of expertise to confirm that it is of an acceptable scientific standard.

# Reviewer report: Reviewer response for version 2

**DOI:** 10.21956/openreseurope.22091.r54416

**Published:** 2025-06-06

**Authors:** Monica Taylor

**Affiliations:** 1 Queensland University of Technology, Brisbane, Queensland, Australia

The revisions and justification for the changes are appropriate.

Where applicable, are recommendations and next steps explained clearly for others to follow? (Please consider whether others in the research community would be able to implement guidelines or recommendations and/or constructively engage in the debate)

Partly

Does the article adequately reference differing views and opinions?

Partly

Are all factual statements correct, and are statements and arguments made adequately supported by citations?

No

Is the rationale for the Open Letter provided in sufficient detail? (Please consider whether existing challenges in the field are outlined clearly and whether the purpose of the letter is explained)

Partly

Is the Open Letter written in accessible language? (Please consider whether all subject-specific terms, concepts and abbreviations are explained)

Yes

Reviewer Expertise:

Access to justice, disaster justice, human rights (in the context of disaster management), climate justice, climate adaptation.

I confirm that I have read this submission and believe that I have an appropriate level of expertise to confirm that it is of an acceptable scientific standard.

# Reviewer report: Reviewer response for version 1

**DOI:** 10.21956/openreseurope.21733.r52894

**Published:** 2025-05-08

**Authors:** Monica Taylor

**Affiliations:** 1 Queensland University of Technology, Brisbane, Queensland, Australia

Thank you for the opportunity to review this open letter entitled, *“Integrating ethical and legal frameworks in multi hazard disaster risk management.”*  As it is an open Letter not a research article, it therefore has a different focus. I provide this feedback based on the criteria and guidelines for reviewing an open letter.

1. Is the rationale for the Open Letter provided in sufficient detail?

The rationale for the open letter was not fully apparent to me in the abstract or the document itself. The objective of the open letter appears to be to critically evaluate the success of an integrated Decision Support System (DSS) developed by the European MEDiate project. But at various stages when reading the article I felt that it did not provide much of a critical lens, and was instead written in a descriptive and promotional style to explain the benefits of the DSS system. I also wasn't sure about the author's standpoint and whether they had some involvement in the creation of the DSS system that is the subject of the article.

 My main critical and constructive feedback relates to the structure of the article. I think it could benefit from a restructure that brings forward the literature review sections, which are provided in the latter part of the paper (part 5). Then a more coherent structure might be: (a) identify the problem or issue under consideration; (b) state a methodology for critical analysis, informed by the literature review, and then (c) apply the literature to the case study (DSS system) to determine whether or not it is meeting the objectives, namely, the integration of ethical and human rights frameworks into multi-hazard DRM. This is where the differing national frameworks and approaches can be introduced, together with key challenges and emerging trends.

 2. Does the article adequately reference differing views and opinions?

 It was very interesting to read about the different national frameworks in MEDiate testbed countries. This was an enjoyable part of the paper that gives context to its overall objective. However, again to structure, I think introducing the national frameworks after the literature review would foreground the evidence against which the DSS system can be evaluated in relation to the national frameworks provided.

 3. Are all factual statements correct, and are statements and arguments made adequately supported by citations?

 For a piece of this length that draws upon a large body of disaster risk management literature, it is considerably under-cited. Citations are often not provided where they ought to be.  An example is on page 8 in part 5.2, where the author states, “Studies highlight that many failures in disaster coordination stem from different agencies and systems being unable to seamlessly share data and models.”  A statement such as this requires a citation to point the reader to the evidence (the studies) that it refers to.  At this stage I do not consider that this article satisfies the criteria that all factual statements are correct and adequately supported by citations.

 4. Is the Open Letter written in accessible language?

 Yes, the language is accessible. There is a repetitive paragraph on Page 3. The second paragraph in Part 2 almost repeats verbatim the preceding paragraph and should be deleted. There also appears to be a typographical error on page 8 where the Covid-19 pandemic sentence says ‘…international organisations advising against *traying *rights through public health measures.’  Is this meant to say betraying?

 5. Where applicable, are recommendations and next steps explained clearly for others to follow?

 Yes, section 6 offers some suggestions for how MEDiate DSS can become more participatory, and it also identifies future research avenues. Section 6 covers a lot of good analytical ground that could be drawn out earlier in the article, applied to the case study and through the national frameworks, and not left right to the end.

 Overall, this is a very interesting article on an important topic that shows promise. With some restructuring it would achieve more clarity and coherence. Closer attention to citation is also required.

 Thank you for the opportunity to provide feedback.

Where applicable, are recommendations and next steps explained clearly for others to follow? (Please consider whether others in the research community would be able to implement guidelines or recommendations and/or constructively engage in the debate)

Partly

Does the article adequately reference differing views and opinions?

Partly

Are all factual statements correct, and are statements and arguments made adequately supported by citations?

No

Is the rationale for the Open Letter provided in sufficient detail? (Please consider whether existing challenges in the field are outlined clearly and whether the purpose of the letter is explained)

Partly

Is the Open Letter written in accessible language? (Please consider whether all subject-specific terms, concepts and abbreviations are explained)

Yes

Reviewer Expertise:

Access to justice, disaster justice, human rights (in the context of disaster management), climate justice, climate adaptation.

I confirm that I have read this submission and believe that I have an appropriate level of expertise to confirm that it is of an acceptable scientific standard, however I have significant reservations, as outlined above.

# Reviewer report: Reviewer response for version 1

**DOI:** 10.21956/openreseurope.21733.r52895

**Published:** 2025-05-08

**Authors:** Matthew Scott

**Affiliations:** 1 Faculty of Law, Lund University, Lund, Skåne County, Sweden; 2 The Raoul Wallenberg Institute of Human Rights and Humanitarian Law, Lund, Sweden

This Open Letter introduces the European MEDiate project, which ‘aims to develop an integrated Decision Support System (DSS) that effectively captures multi-hazard dynamics, emphasizing ethical compliance, fundamental human rights and participatory engagement.’ Any initiative that takes the integration of human rights into disaster risk reduction and management (DRRM) seriously should be commended from the outset. This is, as the author observes, a guiding principle under the Sendai Framework for Disaster Risk Reduction (SFDRR). However, systematic integration of human rights standards and guidelines into DRRM is far from widespread, notwithstanding ten years since the adoption of the SFDRR. Thus, this Open Letter that carefully considers important human rights dimensions of a DRRM system that will operate from local to regional levels across Europe is timely, relevant, interesting and certainly appropriate for indexing. 

 The Open Letter’s strengths are, to some extents, also its limitations. Specifically, although promising to provide ‘a comprehensive examination of the legal frameworks and fundamental rights considerations underpinning the development of the MEDiate DSS’, the Open Letter focuses overwhelmingly on privacy and data protection (Articles 7 and 8 of the Charter on Fundamental Rights), with limited treatment of the right to freedom of movement (Article 45 of the Charter). Other rights protected under EU law are also relevant and the Open Letter (as well as the MEDiate DSS project) would benefit from closer consideration of the non-discrimination obligation, the right to life, the right to an adequate standard of living, the right to the highest attainable standard of physical and mental health, the rights of the child, the rights of persons with disabilities, amongst others. Although the Charter does not specifically protect all of these rights, other instruments guiding the conduct of EU Member States, like the European Social Charter and international human rights law treaties, do. To be fair, the actual promise of the Open Letter is to focus on the rights ‘underpinning the development of the MEDiate DSS’, meaning the limited focus on Articles 7, 8 and 45 may be justified. However, there is more to a human rights-based approach to DRRM than the Open Letter reveals.

 Helpfully, the Open Letter identifies the Framework for Integrating Rights and Equality (FIRE) as an example of how human rights can effectively be integrated into DRRM, including in contexts like risk assessment, risk-sensitive land use planning and emergency preparedness for response. This Framework, developed under the Building Resilience through Inclusive and Climate-Adaptative Disaster Risk Reduction in the Asia-Pacific (BRDR) programme, reflects the outcome of a systematic consolidation of international normative and operational standards and guidelines, including General Comments from UN Treaty Monitoring Bodies and standards like those contained in the Sphere Handbook and the Inter-Agency Standing Committee’s Operational Guidelines on the Protection of Persons in Situations of Natural Disasters. The six-dimensions of the Framework generated through this process include fundamental rights and equality, non-discrimination, participation and access to information, governance systems and structures, social norms and context and agency and empowerment. An associated handbook contains specific standards reflecting a synthesis of international law and operational guidelines. I have included a DOI for the final article describing the FIRE Framework in Climate and Development (Scott M, et al., 2023 [Ref 1]). More information about how the FIRE Framework has been used in other contexts, including pandemic preparedness and response and urban disaster displacement, is available at https://rwi.lu.se/fire. For the Open Letter and the MEDiate initiative to achieve its ambition of integrating fundamental rights into DRRM across the EU, a broader range of rights and associated standards should be taken into account. In terms of recommendations for the Open Letter, it might be helpful to highlight that the rights addressed in the Open Letter and this preliminary phase of MEDiate are those reflected in Articles 7, 8 and 45, but other rights also need to be taken into account as the system grows towards its stated rights-based ambition. 

 The reason I say that the focus on Articles 7, 8 and 45 are also strengths of the paper is because this is the first time I have read such a careful consideration of data protection and privacy principles in a text on DRRM, primarily through the GDPR. Although treatment of Article 45 is not extensive, it is also highly relevant in this context and could even be further developed. One relevant context when it comes to DSS is the tension that can exist between the right to freedom of movement and the duty of the state to protect life. At what point can authorities require people to evacuate (or not return). This question is not conclusively resolved. Even if annotations to the UN Guiding Principles on Internal Displacement suggest enforced evacuations will not be ‘arbitrary’ where there is an imminent risk, more recent scholarship by Burson B, et al. (2018) [Ref 2] provides a more nuanced perspective .

 Taking the Open Letter on its face, there are a small number of other points that the author might wish to address. 

 First, numerous propositions of fact ought to be supported with appropriate references. The lack of references is a weakness of the paper because it makes a critical reader wonder about the reliability of the claim, and also prevents the curious reader from finding out more by following the sources. 

 Second, readability would be improved by addressing repetitions throughout the text. Section 2 is particularly in need of correction in this respect because the two paragraphs in the section essentially present the same message in almost identical wording. Less significant examples concern the repetition of words in a sentence, such as the use of ‘increasing’ twice in the first sentence in the introduction section, the use of ‘collaborative’ and ‘collaborations’ in the last sentence of the first section, amongst others.

 Third, although the Open Letter succeeds in presenting important considerations relating to fundamental rights, ethics, trust and other dimensions of a successful DSS for multi-hazard risk assessment and disaster response, I am left at the end of the Open Letter still trying to visualize what this system actually looks like and how it operates (or is intended to operate) in practice. I appreciate that there is an element of multi-hazard early warning that consolidates different sources of information and can effectively communicate risk information to the public, including through an automated decision-making process. I have less clarity on how the system actually supports decision-making in contexts like evacuation. The Open Letter references evacuations 6 times. It is clear from these references that the DSS can help in planning evacuation routes because it can integrate transport and other relevant data sources. However, it is less clear how the system actually supports ‘ethically guided decisions on evacuations’ [see section 3.2.3 on the UK]. It is highly likely that sensitive personal data about vulnerabilities relating to disability, health status, age etc could contribute to this kind of decision-making, but the mere computerized aggregation of vulnerability characteristics (in accordance with GDPR) would only be a first step in identifying potential support needs in the context of evacuation. Who makes what kind of decisions about ordering, implementing and monitoring evacuations engages critical human rights considerations beyond data protection that the Open Letter might highlight, even if at this stage the MEDiate DSS has not attained that level of rights-based granularity. This level of decision-making has immediate right to life implications, as well as rights implications relating to the specific rights of persons with disabilities and economic and social rights considerations concerning conditions in places where people are evacuated to. Indeed, (automated?) decisions about where people in situations of vulnerability are evacuated to have potentially serious implications relating to the prohibition of torture and inhuman and degrading treatment (Article 4 of the Charter), as well as the right to physical and moral integrity that the European Court of Human Rights has found to be part of the right to a private life under Article 8 of the European Convention on Human Rights (Article 7 of the Charter). Since the Open Letter states ‘these rights provide a lens to evaluate its [the DSS] design and use’, recognition of the wider rights implications of the use of the DSS in the context of evacuations seems warranted.

 Related to this third point, the Open Letter might also clarify the extent to which the DSS includes both fully automated as well as computer-assisted decision-making, and how rights considerations might influence design in this regard.

 I appreciate that the author might not want to address the full range of points I make above, but I do think there could be some more clarity, probably in Section 2, about how the system currently works, how it is expected to work in the future, including specifically the kinds of decisions that are or will be fully automated and which will require humans in the loop. 

 Fourth, some terminology might want to be revisited. In particular, a rights-based approach invites more agentic terminology such as ‘persons with disabilities’ instead of ‘disabled persons’ and ‘survivors/people in situations of vulnerability’ as a more empowering and context-specific alternative to ‘vulnerable survivors’ or ‘the vulnerable’. 

 To conclude, this Open Letter introduces an inspiring initiative that sets out to integrate human rights into DRRM through a data-driven DSS that can support planning, preparedness and response from local to EU-levels. The promise of such an approach is, however, only partly communicated in the Open Letter because the focus is narrowly on data protection, with notable but still limited discussion of non-discrimination, participation and access to information, and lack of reference to critical additional rights such as the right to life, economic, social and cultural rights, and group-specific rights like the rights of the child. Some terminology could be improved to better reflect a rights-based approach, and readers would benefit from a more detailed overview of how the DSS actually works (or is intended to work in the future), plus more references to assist critical and curious readers.

Where applicable, are recommendations and next steps explained clearly for others to follow? (Please consider whether others in the research community would be able to implement guidelines or recommendations and/or constructively engage in the debate)

Yes

Does the article adequately reference differing views and opinions?

Yes

Are all factual statements correct, and are statements and arguments made adequately supported by citations?

No

Is the rationale for the Open Letter provided in sufficient detail? (Please consider whether existing challenges in the field are outlined clearly and whether the purpose of the letter is explained)

Yes

Is the Open Letter written in accessible language? (Please consider whether all subject-specific terms, concepts and abbreviations are explained)

Yes

Reviewer Expertise:

International human rights law; human rights-based approaches to climate change adaptation and disaster risk reduction and management; all forms of environmental human mobility, including evacuation, planned relocation, displacement, forced evictions, resettlement, etc.

I confirm that I have read this submission and believe that I have an appropriate level of expertise to confirm that it is of an acceptable scientific standard, however I have significant reservations, as outlined above.

## References

[1] : FIRE: A framework for integrating human rights and gender equality in disaster risk reduction and climate change adaptation. *Climate and Development* .2023;15(7) : 10.1080/17565529.2022.2138694 622-627 10.1080/17565529.2022.2138694

[2] : The Duty to Move People Out of Harm’s Way in the Context of Climate Change and Disasters. *Refugee Survey Quarterly* .2018;37(4) : 10.1093/rsq/hdy015 379-407 10.1093/rsq/hdy015

# Response

**Published:** 2025-05-09

**Authors:** Zia Lennard

**Affiliations:** R2M Solution, France

Dear Matthew, Thank you very much for your thoughtful and detailed review, which I found immensely constructive. I have carefully addressed your comments as follows:

- **Wider Rights Framework**: I acknowledged that the Open Letter focuses primarily on Articles 7, 8, and 45 of the Charter but also inserted a clear new sentence explicitly stating that broader rights (such as the right to life, non-discrimination, rights of persons with disabilities, children’s rights, etc.) must be integrated as MEDiate evolves. I also inserted a new paragraph on the FIRE Framework (Scott et al., 2023) as a comparative example of integrating a broader set of rights-based standards into DRRM and provided the correct updated citation and URL.
- **Freedom of Movement and Evacuation Ethics**: Following your excellent suggestion, I strengthened the analysis on the tension between evacuation orders and freedom of movement. Specifically, in Section 3.2.3 (United Kingdom testbed), I inserted a new paragraph discussing how the MEDiate DSS includes safeguards (access controls, human-in-the-loop oversight) when processing personal data for evacuation decisions, and how automation must not obscure the ethical stakes involved, such as the rights of persons with disabilities under the CRPD.
- **Supporting References**: I systematically reviewed the text and inserted numerous additional citations where previously assertions were unreferenced, particularly in the literature review and case study sections.
- **Repetitions and Readability**: I removed the repeated paragraph in Section 2 and edited for tighter, more readable language elsewhere (e.g., eliminating the repetitive use of "increasing" and "collaborative collaborations").
- **Clarifying DSS Operation**: I added content in Section 2 to clarify that the current MEDiate DSS operates via computer-assisted decision support with human oversight but anticipates potential future fully automated pathways—carefully explaining the implications for rights protection. The ethical oversight mechanisms for automated decision-making are now clearly spelled out.
- **Terminology**: I revised terminology throughout the paper to reflect person-first, empowering language in line with rights-based standards (e.g., "persons with disabilities" instead of "disabled persons", "survivors/people in situations of vulnerability" instead of "the vulnerable").

Once again, I sincerely thank you for your generous and expert guidance. Your comments significantly improved the scope, precision, and coherence of the revised Open Letter.
